# *Eucommiae cortex* polysaccharides mitigate obesogenic diet-induced cognitive and social dysfunction via modulation of gut microbiota and tryptophan metabolism

**DOI:** 10.7150/thno.72756

**Published:** 2022-05-01

**Authors:** Penghao Sun, Mengli Wang, Zhuoni Li, Jingjing Wei, Feng Liu, Wei Zheng, Xiaoyan Zhu, Xuejun Chai, Shanting Zhao

**Affiliations:** 1 College of Veterinary Medicine, Northwest A&F University, Yangling, China; 2 College of Resources and Environment Sciences, Northwest A&F University, Yangling, China; 3 College of Basic Medicine, Xi'an Medical University, Xi'an, China

**Keywords:** *Eucommiae cortex* polysaccharides, obesogenic diet, gut microbiota, amino acid metabolism, adult neurogenesis

## Abstract

**Rationale:** The high fat and sucrose diet, known as the obesogenic diet (OD), has been related to low-grade chronic inflammation and neurodevelopmental disorders. Emerging evidence suggests that OD influences cognitive and social function via the gut-brain axis. However, the effects of OD during adolescence on future health have been unclear. Meanwhile, the underlying mechanisms and effective interventions are not fully understood. Polysaccharides, one of the most abundant substances in the *Eucommiae cortex*, exhibit potential immunomodulatory and neuroprotective effects. Here, we aimed to investigate the impact of OD on adolescents, explore the modulating roles of *Eucommiae cortex* polysaccharides (EPs) on OD-induced behavioral dysfunction, and elucidate the underlying molecular mechanisms.

**Methods:** In the present study, four-week-old mice were fed with OD for four weeks to simulate persistent OD in adolescents. The behavioral features were accessed by open field test and Morris water maze. The gut bacterial structure was identified by 16S rRNA gene amplicon sequencing. The gene and protein expression in colonic tissues and hippocampus were detected by qRT-PCR, immunoblotting, enzyme-linked immunosorbent assay, and immunofluorescence staining. Detection of biological metabolites in serum and hippocampal tissues was performed by widely targeted metabolomics and targeted metabolomics.

**Results**: We found that OD-fed mice showed cognitive and social-behavioral deficits accompanied by gut dysbiosis and systematic tryptophan (Trp) metabolism disorders, which increased kynurenine (Kyn) concentration in the hippocampus. Bacteria-derived lipopolysaccharide (LPS, endotoxin) induced microglia-mediated neuroinflammation, directing the metabolism of Kyn in the hippocampus toward quinolinic acid (QA), which led to glutamate-mediated hyperactivation of mossy cells (MCs) in hippocampal hilus. Furthermore, OD impaired parvalbumin (PV) interneurons-related local circuits in the hippocampal granule cell layer. These resulted in hippocampal neurogenesis deficits and related behavioral dysfunction in mice. EPs supplementation ameliorated OD-induced gut dysbiosis, as evidenced by inhibiting the expansion of *Escherichia coli* (*E.coli*) and reducing the concentration of LPS in colonic contents and serum, thereby inhibiting the subsequent neuroinflammation. In addition, oral EPs suppressed the peripheral Kyn pathway to reduce the concentration of QA and glutamic acid in the hippocampus of OD-fed mice, thereby rescuing the glutamic acid-triggered neuroexcitotoxicity. These contributed to remodeling the rhythm of hippocampal neurogenesis and mitigated behavioral dysfunction in OD-fed mice.

**Conclusions**: The present study addresses a gap in the understanding of neuronal dysfunction associated with OD during adolescence and provides the first evidence that EPs improved cognitive and social behavior via modulation of gut microbiota and tryptophan metabolism in adolescent mice fed with OD, which may represent novel preemptive therapy for neurodevelopmental disorders via manipulation of the tryptophan metabolite.

## Background

One of the lifestyle changes in Westernized societies over the last decades has been the increased consumption of obesogenic diet (OD), a common diet characterized by high levels of sucrose, saturated fatty acids, and low levels of dietary fiber, which is implicated in various metabolic diseases 1, 2. The gut is the largest mucosal immune system and requires a sustained barrier and modulatory mechanisms to sustain tissue homeostasis, as well as overall human physiology 3. Disturbed gut microbiota compromises barrier integrity and triggers systemic inflammatory responses 4. Within the range of factors, dietary habits exert the most remarkable impact on the diversity and composition of gut microbiota 5. Based on experimental reports from human studies and animal models, it has been established that OD can cause gut dysbiosis 6 and contribute to chronic inflammation, exacerbating the severity of inflammatory diseases 7, 8. Researchers prove that merely transplanting the fecal microbiota from OD-fed mice to conventional mice with a standard diet was sufficient to cause obesity-related symptoms, including weight gain, metabolic disorders, and systemic inflammation 9, 10. These results highlight the initiating role of gut microbiota in OD-induced inflammation-associated impairments.

During adolescence, the last developmental stage before adulthood, the brain responds strongly to environmental stimuli, which will alter neuronal structure and affect the maturation of the individual's social behavior and cognitive abilities, making it a vulnerable period for the onset of psychosis 11. Pre-early inflammatory events are known to cause persistent negative effects on central nervous system (CNS) development and, therefore, on future behavior 12. Hippocampal inflammation during brain development has been proven to dramatically impair individuals' behavior in adulthood 13. Recent studies reveal that inflammatory events occurring at developmental stages cause CNS dysfunction via processes such as microglial activity and adult neurogenesis 14. In contrast to the highly diverse and stable gut microbiota of adults, the gut microbial composition of adolescents is usually simple and more susceptible to disruptions 15, 16. However, few studies have been published on the effects of OD during adolescence on future health.

*Eucommia ulmoides oliver* is a unique tree species in China. *Eucommiae cortex* has been used as a dietary supplement for nearly two thousand years 17. Polysaccharides are biological macromolecules widely found in the daily diet and consist of more than a dozen monosaccharides linked together by glycosidic bonds. *Eucommiae cortex* polysaccharides (EPs) have been shown to act as prebiotics possessing neuroprotection and anti-inflammatory activities 18. Recent studies have shown that polysaccharides can exert a beneficial modulatory effect on the gut microbiota, in particular leading to the reduced abundance of pathogens, the increased population of favorable bacteria and the production of short-chain fatty acids (SCFAs), which supports the health of the host 5. However, different sources of polysaccharides present distinct structural types and have diverse influences on the intestinal microbiota 19. For instance, oat β-glucan enhances the abundance of *Clostridium* and *Butyricoccus* whereas inhibiting the growth of *Bacteroides*, *Lactobacillus*, *Oscillospira,* and *Ruminococcus* 20. Arabinoxylan boosts the growth of *Bifidobacterium*, *Lactobacillus*, and *Bacteroides*, as well as lowers the relative abundance of *Fusobacterium*, *Bilophila,* and *Desulfovibrio* 21. Different microorganisms in the gut possess specific enzymes, regulatory and transport mechanisms and therefore specialize in decomposing various polysaccharides 19. The effects of EPs on the intestinal microbiota have not been well established.

In the present study, four-week-old mice fed with OD for four weeks, simulating persistent OD in adolescents, exhibited cognitive and social behavioral deficits. We demonstrated that EPs supplementation effectively inhibited gut dysbiosis, thereby reducing serum endotoxin and subsequent neuroinflammation. Moreover, EPs supplementation attenuated OD-induced metabolic syndrome and reduced the concentration of quinolinic acid (QA) and glutamic acid in the hippocampus. These contributed to rescuing adult neurogenesis defects and behavioral anomalies in OD-fed mice. This study addresses a gap in understanding neuronal dysfunction associated with OD during adolescence and indicates EPs may be used as prebiotic agents to prevent OD-related neurodevelopmental disorders.

## Methods

### Preparation of EPs

The *Eucommiae cortex* was purchased from Tong Ren Tang company, China, and the extraction and purification of polysaccharides were processed as described before 22.

### Determination of monosaccharide and molecular weight of EPs

The determination of EPs properties, including monosaccharide composition and molecular weight, was examined by Beijing ZKGX Research Institute of Chemical Technology (Beijing, China).

### Mice, diets, and experimental setup

Male ICR mice were obtained from the SPF Biotechnology Co., Ltd. (Beijing, China). Mice were housed in an animal facility under standard conditions (12-hour light/dark cycle, humidity of 50 ± 15%, and temperature of 22 ± 2°C). Male mice aged four weeks were placed into three groups of twenty animals each at random. Standard pelleted diets as well as obesogenic diet were purchased from Jiangsu Xietong Medicine Bioengineering Co., Jiangsu, China, and kept at -20 °C for the duration of the study. Mice were housed in cages with three or four animals per cage and provided the following diet: control diet (Chow; XT079B-C), obesogenic diet (OD; XT079B) (high fat, high sucrose, containing 0.15% cholesterol), and an obesogenic diet with EPs supplementation (EPs: obesogenic diet plus daily supplementation of purified EPs (400mg/kg) by intragastric gavage). Animal janitors and investigators performing the experiments were blinded to the group assignment of mice during the experiment. The Guide for the Care and Use of Laboratory Animals: Eighth Edition was used to carry out all experiments. We followed all related animal testing ethics regulations, as well as the studies and protocols approved by the Ethics Committee of the College of Veterinary Medicine, Northwest A&F University.

### Fecal sample DNA extraction and PCR amplification and sequencing

Colon contents were collected under sterile conditions, and all samples were frozen at -80 °C before DNA extraction and analysis. QIAamp DNA Stool Mini Kit (QIAGEN, Germany) was used to extract fecal bacterial DNA, and all procedures were based on the manufacturer's instructions. The V3-V4 hypervariable regions of the bacterial 16S rRNA gene was amplified with primers 341 F (5'-CCTAYGGGRBGCASCAG -3') and 806 R (5'-GGACTACNNGGGT ATCTAAT-3'). The sequencing steps were conducted by Magigene Technology Company(Guangzhou, China).

### Processing of sequencing data

Raw bacterial 16S rRNA gene sequence data were generated by Illumina Miseq PE250. After truncating barcodes and primers, the sequence data were imported into Quantitative Insights Into Microbial Ecology2 (QIIIME2, version 2019.10) 23 platform for further analysis. Divisive Amplicon Denoising Algorithm 2 (DADA2) based on the phyloseq 24 package was applied for these sequences denoising, generating representative sequence and amplicon sequence variants (ASVs) table. The taxonomy of each representative 16S bacterial gene sequence was analyzed by the RDP Bayes-Classifier 25 using a confidence threshold of 80%.

### *E.coli* population of colon contents

The expansion of *E.coli* was tested by *E.coli* chromogenic medium from Hopebio (HB7001) and all procedures were based on the manufacturer's instructions.

### Metabonomics analysis

Serum and hippocampal tissue and colonic contents were collected at the end of the experiment and were stored at -80 °C until analysis. The samples were delivered to Metware Biotechnology CO., Ltd. (Wuhan, Chian) to perform metabonomics analysis.

### Protein Analysis and Immunofluorescence

The primary antibodies, including indoleamine 2, 3 dioxygenase1 (IDO1), ionized calcium binding adapter molecule 1 (Iba1), glial fibrillary acidic protein (GFAP), T-brain gene-2 (Tbr2), doublecortin (DCX), Prox1, act-casp3, PV, c-Fos, Calretinin, occluding, β-actin, toll-like receptor 4 (TLR4), and NF-κB, were purchased from Abcam, Invitrogen, Santa Cruz Biotechnology, and Cell Signaling Technology companies. The western blot and immunofluorescence procedures were performed following our previous protocols with minor modifications 26. The modification is mainly the adjustment of the antibody dilution.

### RNA extraction and qRT-PCR

RNA extraction and qRT-PCR analysis were performed as our previous protocols 27 and using primer as following: GAPDH (forward 5'- AGGTTGTCTCCTGCGACTGCA, reverse 5'- GTGGTCCAGGGTTTCTTACTCC), TNF-α (forward 5'- AGTCCGGGCAGGTCTACTTT, reverse 5'-GTCACTGTCCCAGCATCTTGT), MCP-1 (forward 5'- TCACTGAAGCCAGCTCTCTCT, reverse 5'-GTGGGGCGTTAACTGCAT), IL-1β (forward 5'- TGACGGACCCCAAAAGATGA, reverse 5'-TCTCCACAGCCACAATGAGT), iNOS (forward 5'- GAGCGAGTTGTGGATTGTC, reverse 5'- CCAGGAAGTAGGTGAGGG), IL-6 (forward 5'- ACCGCTATGAAGTTCCTCTC, reverse 5'-CTCTGTGAAGTCTCCTCTCC).

### Behavioral Test

The behavioral tests, including the open field test and Morris water maze, were performed following our previous protocols with some modifications 28. To be specific, the detection time was extended to 30 min in the open field test. Data analysis strategies refer to previous studies^29^. To avoid experimental error due to latent variables, mice participating in the Morris water maze were not subjected to subsequent experimental analysis.

### Statistical analysis

A rarefied ASVs table with 25582 reads per sample was constructed for the 16S rRNA gene amplicon sequencing data in order to quantify bacterial diversity based on the minimal number of sequences per sample processed by QIIME2 23. On the rarefied ASVs table, a performing principal coordinates analysis (PCoA) based on Bray-Curtis dissimilarity was done. For bacterial diversity, the PERMANOVA test was used as a significant test. The Mann-Whitney U-test was used to identify the indicator taxa. The repeated-measures ANOVA was used to assess the behavioral data from the open field test. Weighted gene co-expression network analysis (WGCNA) was performed to define trends in metabolites co-expression 30. For multiple comparisons, one-way ANOVA was performed using R (version 4.03) software and Dunnett's test is adopted for post-hoc testing. Details of each test including post-hoc assessment are specified in figure legends. *P* < 0.05 was used to evaluate significance.

## Results

### Morphology, monosaccharide, and molecular weight of EPs

To ensure the safety of the extracted polysaccharides, a green and effective three-phase partitioning technique was used to purify the EPs 22. The typical micrograph of EPs was presented in Figure S1. Purified EPs were granular and/or fragmented and exhibited a rough surface with pores and crevices. The size distribution of EPs was not homogeneous. The monosaccharide composition of EPs was shown in Figure S2 and Table S1. The molecular weight of purified EPs ranged from 630-251000 Da (Figure S3).

### EPs mitigated OD-induced obesity-related symptoms and behavioral dysfunction

**Figure 1:**
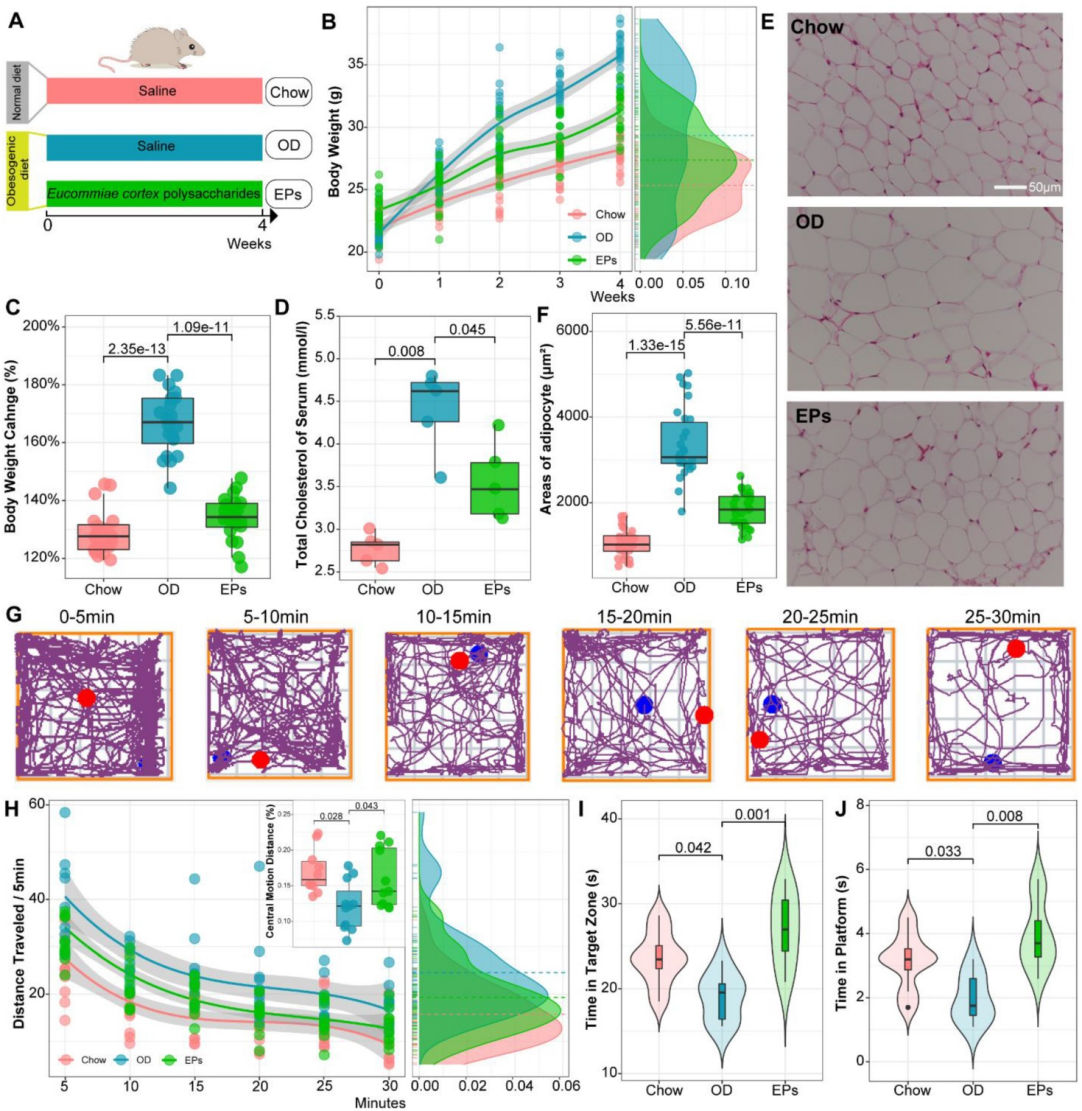
** EPs mitigated OD-induced obesity-related symptoms and behavioral dysfunction.** (A) The schematic diagram for time and administration in the experimental procedure. Mice were fed with the following diet respectively, Chow: normal diet, OD: a diet containing high sucrose and fat, EPs: a diet containing high sucrose and fat, but supplemented with EPs (*n* = 20 individuals/group). (B) Growth curve of body weight during experiment. (C) Quantitative analysis of weight change at the end of the experiment. Obesity traits, including total serum cholesterol (D) and adipocyte size (epididymal white adipose tissue) (E-F), were quantitatively analyzed. (G) Representative tracing of mice in open field test at each 5-min time interval of the 30-min. Chow *n* = 10, OD and EPs *n* = 11. (H) The average distance traveled (meters) was measured in 5-min time bins across a 30-min session in an open field box. (Inset) The ratio of central motion distance of mice in the entire box during 30-min. (I, J) Morris water maze test (*n* = 8 individuals/group). Compared to Chow, the mice fed with OD spent less time in the target zone and platform area, indicating the deficits of spatial learning and memory, which were rescued by EPs supplementation. In D, *n* = 5 individuals/group. In F, *n* = 30 slices from 6 mice. In B, smoothing curves based on the linear model are shown in gray with 95% confidence intervals. In C-D, F, and H-J, Chow and EPs were compared to OD by one-way ANOVA, adjusted for multiple comparisons by Dunnett's post-hoc. In H, repeated-measures ANOVA was used to assess the adaptation of mice to novel environments. Lines in boxes represent median, top and bottom of boxes represent first and third quartiles, and whiskers represent 1.5 interquartile range.

To simulate consistent OD in adolescents and examine the beneficial effects of EPs, four-week-old mice were fed with OD for four weeks and supplemented with EPs or saline (Figure 1A). Bodyweight, total serum cholesterol, and epididymal fat accumulation were significantly increased in mice fed OD for four weeks compared to mice fed a normal diet (Figure 1B-F). EPs coadministration effectively mitigated OD-induced obesity-related symptoms, including weight gain (Figure 1B-C), elevated serum cholesterol (Figure 1D), and epididymal fat accumulation (Figure 1E-F).

We conducted open field test and Morris water maze to examine the social and cognitive behavior of mice, respectively. To examine the effect of body weight changes on the movement performance of mice, we assessed the locomotor ability and coordination of mice (randomly selected) by rotarod system before performing behavioral studies. Our results showed that weight change showed no significant effect on motility and coordination in mice (Figure S4). Mice were placed in a novel, open-field activity box to investigate their spontaneous motor activity measured for 30 min (Figure 1G). OD-fed mice showed greater distance traveled of the open field (Figure 1H). Meanwhile, a significant difference between groups was detected in habituation over time (repeated measures ANOVA: Chow VS OD, *P* < 0.000; OD VS EPs, *P* < 0.005). These results indicated that sustained OD during adolescence decreased the adaptation of mice to the novel environment. OD-fed mice supplemented with EPs exhibited a more similar pattern of social behavior to chow-fed mice (Figure 1H). In addition, OD-fed mice exhibited lower central motion distance relative to normal diet mice, indicating that OD during adolescence remarkably interfered with exploratory behavior in mice, and this defect was alleviated by EPs supplementation (Figure 1H). We further investigated the effect of OD on cognitive behaviors in adolescent mice. With the Morris water maze test, OD-fed mice showed deficits in hippocampus-dependent spatial learning and memory (Figure 1I-J). Supplementation with EPs significantly improved cognition (Figure 1I-J). These results indicate that EPs supplementation mitigated OD-induced behavioral dysfunction.

### EPs reshaped the gut microbiota and alleviated the subsequent experimental colitis in OD-fed mice

**Figure 2:**
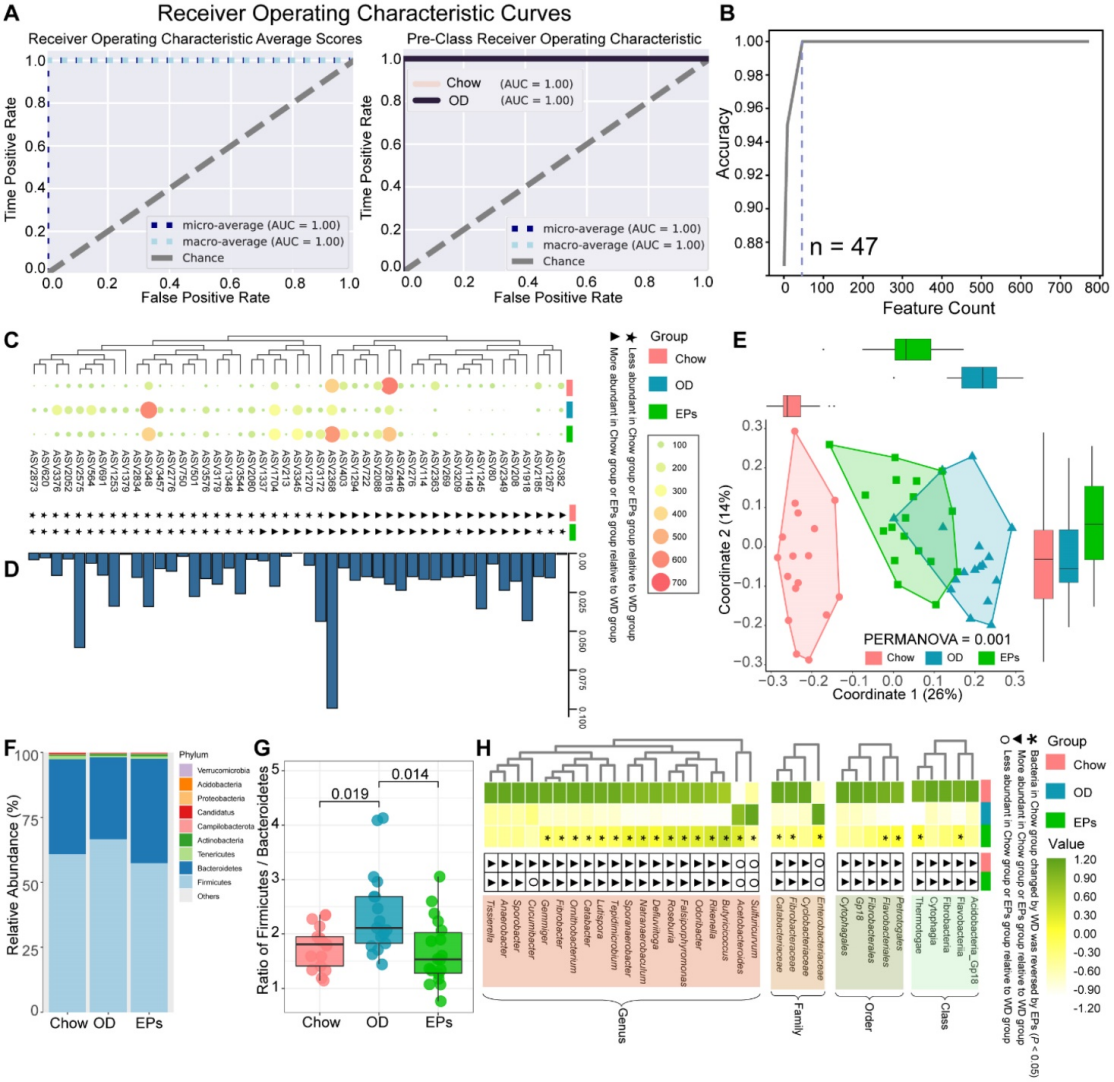
** EPs remodeled the gut microbiota in OD-fed mice.** (A) Receiver operating characteristic curve is a graphical representation of the classification accuracy of a machine-learning model (Chow and OD *n* = 17, EPs *n* = 18). The 5-fold cross-validation method was adopted to train the classification model to identify the indicator taxa between the Chow and OD groups. The trained classification model accurately distinguished the type of gut fecal samples from their original groups. (B) 47 features were selected to construct a classification model. The abundance of ASVs selected to construct classification models was shown in C. (D) The importance of ASVs in C was calculated. (E) ASV-based PCoA with Bray-Curtis distance (for principal component1 and principal component2) showed the variations of gut microbial β-diversity of three groups and assessed by PERMANOVA. (F) Bacterial taxonomic profiling at the phylum level from different groups. (G) Firmicutes/Bacteroidetes ratio in the indicated groups. (H) Heatmap showed the abundance of taxa significantly influenced by OD compared with that in mice fed with normal diet. In G, statistical significance compared to OD by one-way ANOVA, adjusted for multiple comparisons by Dunnett's post-hoc. In H, Mann-Whitney U-test was used to identify the bacterial taxa affected by high fat and sucrose diet (Chow VS OD) and to assess the remodeling effect of polysaccharides on diet-disturbed bacteria (OD VS EPs). Lines in boxes represent median, top and bottom of boxes represent first and third quartiles, and whiskers represent 1.5 interquartile range. In L, smoothing curves based on the linear model are shown in gray with 95% confidence intervals.

Microbial communities can be sensitive indicators of environmental change and dysbiosis 31. As the initiating role of gut dysbiosis in OD-induced symptoms, we next performed 16S rRNA gene amplicon sequencing to assess the impact of OD on the gut microbiota. Firstly, we performed analysis using the random forest (RF) machine learning method to identify the sensitive bacterial indicator for OD. After randomly separating the 34 samples (Chow: 17; OD: 17) into training (70%) and validation (30%) sets, we trained the RF regressor to identify indicators using a vector of abundances for 3616 ASVs (Figure 2A). This approach identified a collection of gut microbial signatures comprised of 47 discriminatory ASVs (Figure 2B) that accurately distinguished the type of fecal samples (Figure 2A). The abundance of features identified by the RF method was shown in Figure 2C. Meanwhile, the importance of features was assessed according to the permutation feature importance technique (Figure 2D). We further assessed the remodeling effects of EPs on gut microbiota in OD-fed mice by performing PCoA based on 47 ASVs identified by RF. PCoA based on Bray-Curtis distances showed distinct clustering of the gut microbiota among groups (Figure 2E). Interestingly, the microbiota composition of mice in EPs group was more similar to that in mice fed with normal diet (Figure 2E). These results suggest that oral EPs mitigated OD-induced disturbance in the gut microbial community to some extent.

**Figure 3:**
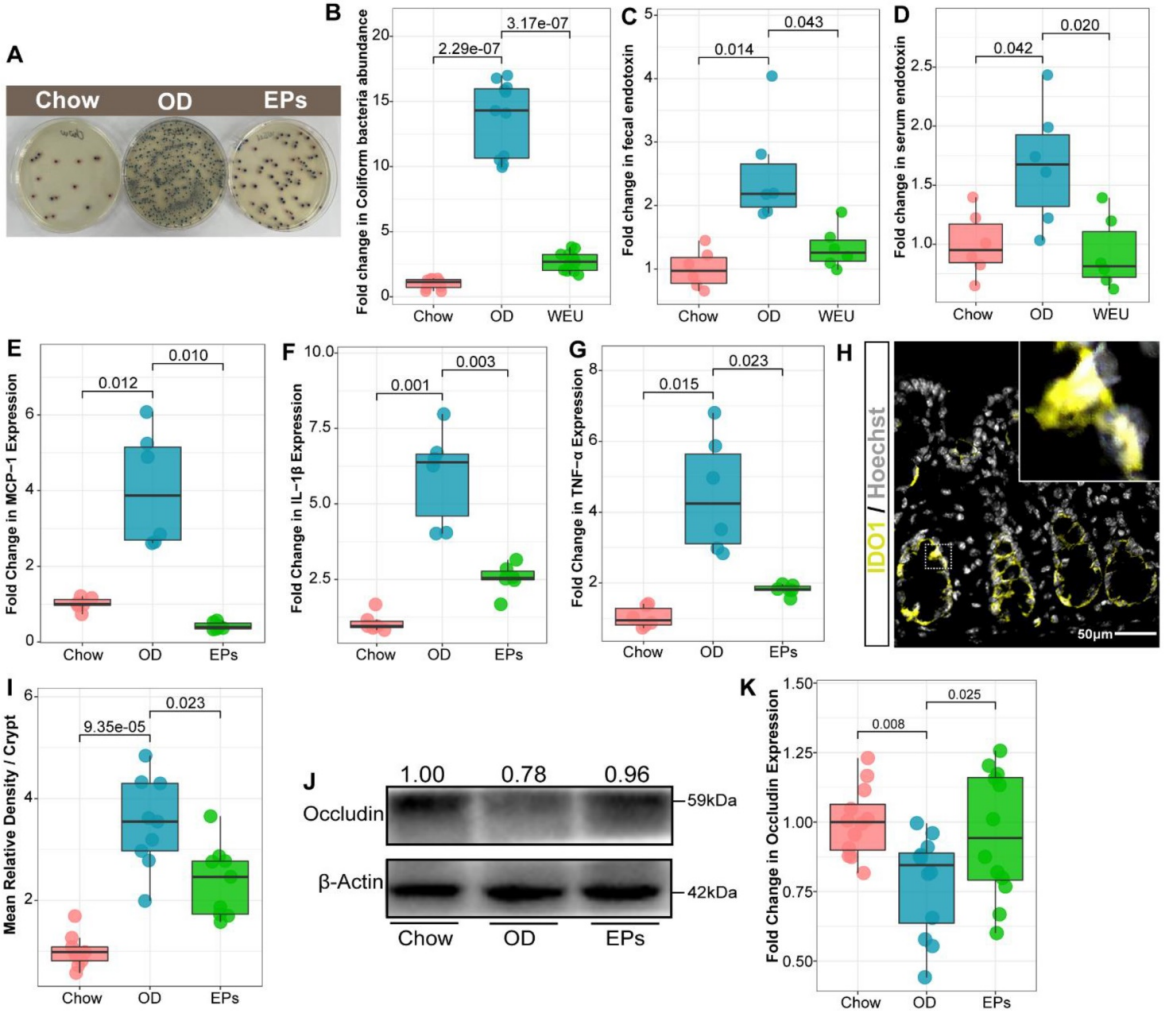
** EPs inhibited the expansion of *E. coli* in the gut and alleviated the subsequent experimental colitis.** (A-B) Chromogenic culture for assessing the abundance of *E. coli* in colonic contents. Supplementation with EPs inhibited the OD-induced expansion of *E. coli* in the colon (*n* = 11 for each group). OD increased the endotoxin concentration in colon contents (C) and serum (D), rescued by supplementation with EPs (*n* = 6 individuals/group). Relative mRNA expression levels of MCP-1 (E), IL-1β (F), and TNF-α (G) in colonic tissues was assessed using qRT-PCR (*n* = 6 individuals/group). (H) Fluorescent immunostaining of IDO1 (yellow) in mouse colonic sections. Nuclei were counterstained with Hoechst (white). (I) The expression of IDO1 was significantly enhanced in OD, which was inhibited by supplementation of EPs (*n* = 9 slices from 3 mice). (J-K) Immunoblot analysis for occludin in colon tissue. Quantification: band intensity normalized to β-actin (*n* = 12 for each group). Statistical significance compared to OD by one-way ANOVA, adjusted for multiple comparisons by Dunnett post-hoc. Lines in boxes represent median, top and bottom of boxes represent first and third quartiles, and whiskers represent 1.5 interquartile range.

Analysis of intestinal microbiology in the obese human shows an elevated Firmicutes-to-Bacteroidetes ratio, as well as an expansion of endotoxin-producing *Enterobacteriaceae* 32, 33. Consistent with the microbial profile of obese humans, OD during adolescence promoted the abundance of Firmicutes and decreased the abundance of Bacteroidetes in mice (Figure 2F), reflecting a significant increase in Firmicutes-to-Bacteroidetes ratio (Figure 2G). Notably, administration of EPs significantly decreased the Firmicutes-to-Bacteroidetes ratio of gut microbiota (Figure 2G). By LDA effect size (LEfSe) analysis, we found that OD significantly reduced the abundance of SCFAs-producing bacteria; including *Butyricicoccus*,* Fibrobacter*, and *Roseburia* (Figure 2H). *Butyricicoccus* 34 and *Roseburia* 35 are the primary butyrate producers in the colon. The gas chromatography-mass spectrometry based approach showed that OD significantly reduced the butyrate concentration and slightly affected the concentration of propionic acid in colon contents (Figure S5A-B). EPs supplementation promoted the growth of SCFAs-producing bacteria, including *Butyricicoccus*,* Fibrobacter*, and *Roseburia*, and enhanced the concentration of SCFAs, especially butyric acid, in the colon (Figure 2H; Figure S5A-B). Healthy caecum/colon microbiota is characterized by a predominance of exclusively anaerobic members that digest complex dietary carbohydrates (such as polysaccharides and fiber) into SCFAs that contribute to gut immune development and restraint pathogens 4, 36, whereas the expansion of the pathogenic *Enterobacteriaceae* is a hallmark of dysbiosis in the intestinal flora 37. As shown in Figure 2H, OD significantly increased the abundance of *Enterobacteriaceae* in the colon. Consistent with the results of 16S rRNA gene amplicon sequencing, chromogenic culture experiments proved that OD during adolescence resulted in the expansion of *E.coli* in the colon (Figure 3A-B)*.* Meanwhile, the enzyme-linked immunosorbent assay showed that bacteria-derived LPS was significantly elevated in the feces and serum of OD-fed mice (Figure 3C-D). EP supplementation remarkably inhibited the expansion of *E.coli* (Figure 2H; Figure 3A-B), and also reduced the concentration of LPS in feces and serum in OD-fed mice (Figure 3C-D).

OD-disrupted gut microbiota and subsequent endotoxin trigger the expression of proinflammatory cytokines in immune cells and contribute to the development of organogenic and systemic inflammation 38. Consistent with reports from adult-phase studies 2, OD during adolescence caused colitis characterized by upregulated mRNA expression of proinflammatory cytokines, including tumor necrosis factor α (TNF-α), interleukin-1β (IL-1β), and monocyte chemoattractant protein-1 (MCP-1), relative to mice on normal diet (Figure 3E-G). Moreover, the expression of IDO1 was upregulated in colon tissues of OD-fed mice (Figure 3H-I). In line with the beneficial effects of EPs on gut dysbiosis, supplementation with EPs effectively suppressed the colonic inflammation of OD-fed mice (Figure 3E-I). Recent researches have revealed that gut dysbiosis impairs the gut barrier, allowing bacterial endotoxins to enter tissues more easily 2, 38. As shown in Figure 3J-K, four-week OD significantly reduced the occludin protein expression in colonic tissue, indicating increased gut permeability, but this impairment was alleviated by EPs coadministration in OD-fed mice. Collectively, these results indicate that gut dysbiosis-related colitis symptoms were ameliorated in OD-fed mice by oral EPs.

### EPs attenuated OD-induced metabolic disruption

**Figure 4:**
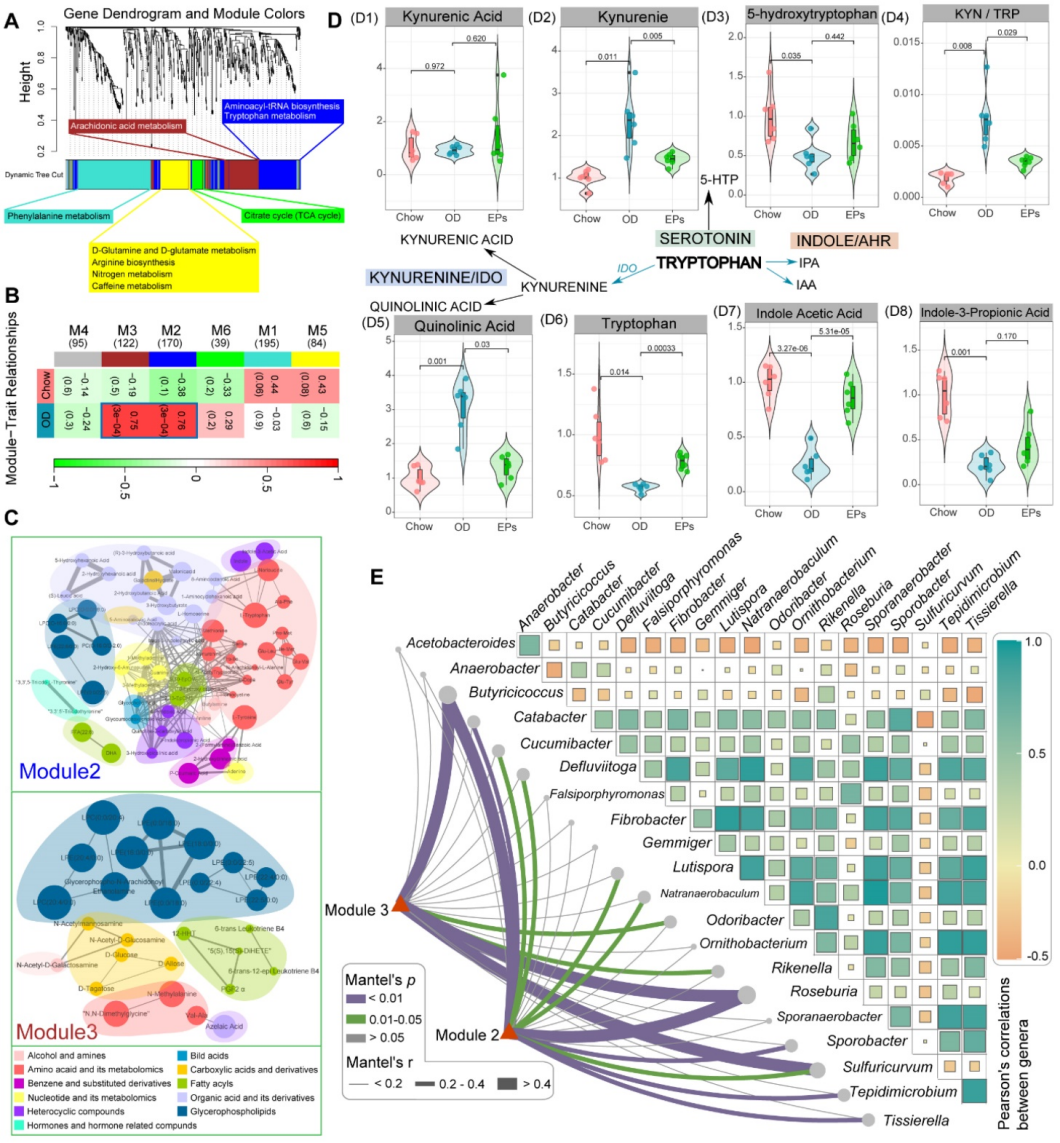
** EPs attenuated OD-induced metabolic disruption.** (A) WGCNA cluster dendrogram groups metabolite into distinct metabolite modules (with different colors) defined by dendrogram branch cutting. The functional enrichment analysis of different modules was performed by MetaboAnalyst. (B) Association of metabolite WGCNA modules with group information. (C) Network diagrams of differential metabolites in two metabolic modules (M2, M3) that are significantly correlated to OD. Circle colors indicate the different class I metabolites category in each module; circle size indicates the abundance of the metabolites. Metabolites were mainly classified into three classes: amino acid and its metabolomics, organic acid and its derivatives, and glycerophospholipids. (D) These serum metabolites were mapped into the tryptophan metabolic pathway. The majority of metabolites in the kynurenine pathway were upregulated in OD group relative to Chow group (D1-2, D5). Meanwhile, serotonin (D3) and indole pathways (D7-8) were depleted in the OD group. (E) Pairwise comparisons of OD-altered genera are shown, with a color gradient denoting Pearson's correlation coefficients. OD-associated metabolite modules (M2, M3) were related to each genus by Mantel tests. Edge width corresponds to the Mantel's r statistic for the corresponding distance correlations, and edge color denotes the statistical significance based on 999 permutations. *n* = 6 individuals/group. Statistical significance compared to OD by one-way ANOVA, adjusted for multiple comparisons by Dunnett post-hoc. Lines in boxes represent median, top and bottom of boxes represent first and third quartiles, and whiskers represent 1.5 interquartile range.

The gut microbiota plays an important role in the physiological homeostasis of the host. Numerous of these effects are mediated through the metabolites derived from microorganisms or are transformed by environmental or host molecules 39. After assessing OD-induced gut dysbiosis, we next wanted to investigate the subsequent changes in systemic metabolites. To this end, we conducted widely targeted metabolomics analyses of serum samples from mice. Co-expression analysis of the systematic metabolic profile effectively associates metabolites into networks related to clinical information and functional variations. The WGCNA method was adopted to define trends in metabolites co-expression. After determining the soft threshold, we set the minimum size of the WGCNA module to 7 and the height of the merged module to 0.25. These parameters divided the metabolites into 6 modules and are ordered by size from M1 (largest, 195 metabolites) to M6 (smallest, 39 metabolites) (Figure 4A-B). Subsequent correlation analysis identified two significant associations between metabolite modules and OD: M2 (*r* = 0.76, *P* < 0.01) and M3 (*r* = 0.75, *P* < 0.01) (Figure 4B). The metabolites of M2 and M3 were mainly classified into three classes: amino acid and its metabolomics, organic acid and its derivatives, and glycerophospholipids (Figure 4C). Functional analysis revealed that the metabolites in OD-associated modules mainly related to aminoacyl-tRNA biosynthesis, tryptophan metabolism, and arachidonic acid metabolism (Figure 4A). After correlating OD-altered genera with the matrix of OD-associated metabolites modules by Mantel-test, we found that butyrate-producing bacteria, *Butyricicoccus* and *Roseburia*, strongly correlated with these metabolite modules (Figure 4E). These results suggest that gut dysbiosis may be a potential risk factor for OD-related metabolic disruption.

Tryptophan (Trp) metabolism disorders are strongly associated with neurodegenerative and psychiatric diseases 40. Trp metabolic pathways are directly or indirectly regulated by the gut microbiota 41. Next, we further checked the variances of serum metabolites mapped into the Trp metabolic pathway. A fraction of dietary Trp was direct transformed by intestinal microbiota into indole and its derivatives 40. The serum of OD-fed mice contained lower concentration of indole acetic acid (IAA) and indole-3-propionic acid (IPA), ligands for aryl hydrocarbon receptor, compared to that of mice fed with normal diet (Figure 4 D7-D8). IAA and IPA are known to affect intestinal permeability and host immunity 42. Supplementation of EPs promoted serum IAA concentrations in OD-fed mice (Figure 4 D7-D8), indicating that EPs reduced permeability and inflammation in colon tissue of OD-fed mice may partly be attributed to modulation of the gut microbiota-mediated indole-pathway of Trp metabolism. Notably, in the serum of OD-fed mice, Trp level was reduced, and the Kyn/Trp ratio was elevated (Figure 4 D4, D6), coinciding with increased expression of IDO1 in colon tissues (Figure 3H-I). The rate-limiting enzyme IDO1 in the gut plays a decisive role in the regulation of the systemic Kyn pathway 43. Oral administration of EPs significantly inhibited the Kyn pathway in OD-fed mice, characterized by reduced serum Kyn and Kyn/Trp ratio (Figure 4 D2, D4). The critical role of the gut microbiota in stimulating IDO1 activity has been clearly demonstrated 41. These results indicate that the ameliorative effect of EPs on the Kyn pathway in OD-fed mice might be due to remodeling gut microbiota, thereby alleviating the upregulation of IDO1 associated with colitis. The serum of OD-fed mice exhibited higher concentration of QA, a selective N-methyl-D-aspartate receptor agonist, but this effect was eliminated by EPs supplementation (Figure 4 D5). These results inspired us that the disruption of Trp metabolism caused by OD may be a potential trigger for the behavioral dysfunction of adolescent mice.

### EPs suppressed the OD-induced hippocampal neuroinflammation

**Figure 5:**
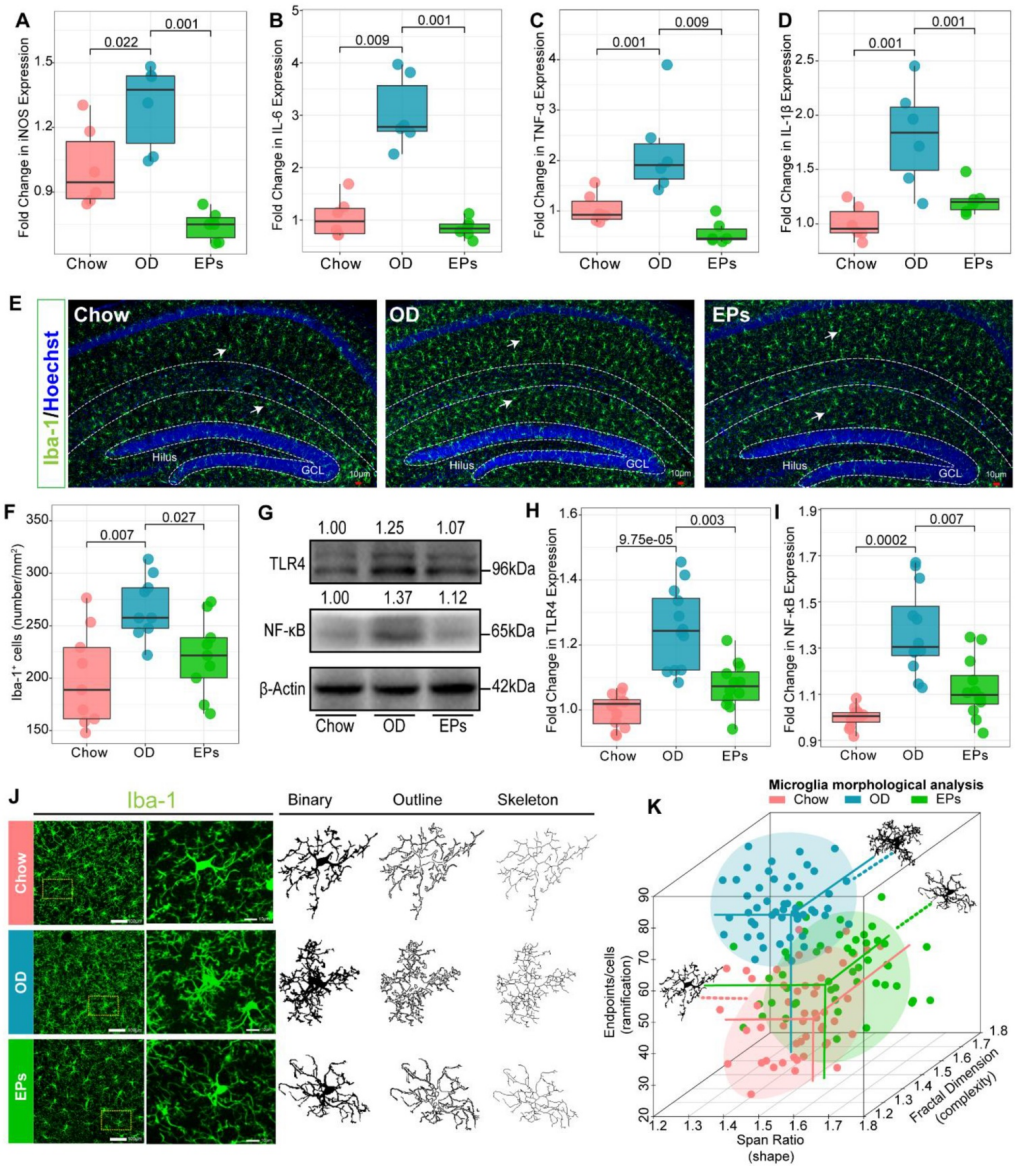
** EPs inhibited OD-induced hippocampal neuroinflammation.** Relative expression of iNOS (A), IL-6 (B), TNF-α (C), and IL-1β (D) in hippocampus tissues was assessed using qRT-PCR (*n* = 6 individuals/group). Compared to those in Chow, the above inflammatory and oxidative factors were significantly upregulated in OD, which was suppressed by supplementation of EPs. (E) Confocal photomicrographs of microglia (green) labeled with antibody against Iba1 in hippocampus. Nuclei were counterstained with Hoechst (blue). (F) Quantitative analysis of microglial density in the molecular layer of dentate gyrus (*n* = 9 slices from 3 mice). OD increased the density of microglia compared to that in Chow. However, supplementation of EPs decreased microglia density induced by OD. (G-I) The expression of TLR4 and NF-κB in hippocampus was examined by using western blot and quantitatively analyzed (*n* = 12 for each group). EPs supplementation decreased the expression of TLR4 and NF-κB in OD-fed mice. (J) Skeleton analysis of microglia morphologies in hippocampus. Original photomicrographs (green) were subjected to a series of uniform ImageJ plugin protocols prior to conversion to binary images; binary images (dark) were then skeletonized. All skeleton analysis was completed on full-sized photomicrographs. (K) Diverse microglia morphologies across three groups (*n* = 50 cells from 3 mice). In OD-fed mice, the microglia showed more and shorter branches than in Chow. The morphology of microglia in EPs was closer to that in Chow. Statistical significance compared to OD group by one-way ANOVA, adjusted for multiple comparisons by Dunnett post-hoc test. Lines in boxes represent median, top and bottom of boxes represent first and third quartiles, and whiskers represent 1.5 interquartile range.

Recent studies have shown that the gut microbiota regulates postnatal neurogenesis and neurological function in the hippocampus by governing the maturation and activation of microglia, the primary immune cells of the CNS 44. Hence, we further investigated the effect of OD on immune homeostasis in the CNS of adolescent mice. A range of neuroinflammatory responses was observed in the hippocampus of OD-fed mice, including significant upregulation of inducible nitric oxide synthase (iNOS), IL-1β, TNF-α, and interleukin-6 (IL-6) (Figure 5A-D; Figure S6A-C). Compared to standard diet-fed mice, the density of microglia (Iba1^+^) throughout the hippocampus was significantly increased in OD-fed mice as revealed by immunofluorescence staining (Figure 5E-F). Consistent with the impacts on gut microbiota, EPs supplementation caused a significant reduction in proinflammatory cytokines, including iNOS, IL-1β, TNF-α, and IL-6, and the density of microglia through the whole hippocampus in OD-fed mice (Figure 5A-F; Figure S6A-C). OD-induced gut dysbiosis resulted in the permeabilization of the gut barrier (Figure 2; Figure 3J-K), which led to the increased passage of bacterial LPS into the circulation (Figure 3D), low-grade endotoxemia, and activation of innate immune cells such as microglia (Figure 5E-F). This mechanism illustrates the direct and early impact of the diet as a source of proinflammatory mediators of CNS 38. Endotoxin and TLR4 signaling regulate the expression of proinflammatory cytokines in specific organs and contribute to persistent inflammation in OD-fed mice 33. Consistent with the inhibitory effect on *E. coli* expansion and LPS production, EPs coadministration down-regulated TLR4 expression in the hippocampus of OD-fed mice (Figure 5G-H). Since the TLR4 signaling pathway induces the production of proinflammatory cytokines through activation of NF-κB, we examined whether this pathway is affected by EPs supplementation. In line with the inhibitory effect on TLR4, EPs suppressed NF-κB protein expression in the hippocampus of OD-fed mice (Figure 5G, I). These results revealed that OD during adolescence resulted in hippocampal neuroinflammation, and the beneficial effect of EPs on hippocampal neuroinflammation may due to reduce the LPS production by inhibiting the expansion of *E.coli*. Due to the context-dependent function of microglia, morphological variation in microglia is commonly used as an indicator to investigate inflammation and dysfunction in the CNS 45. Our results showed that microglia in hippocampus of OD-fed mice exhibited a more complicated fractal dimension and also significantly increased endpoints per cells compared to those in chow-fed mice (Figure 5J-K; Figure S7A, C). Meanwhile, OD significantly reduced the span ratio, a measure of cell shape/elongation, of microglia (Figure 5J-K; Figure S7B). These alterations were consistent with Figure 5A-D, suggesting significant polarization of microglia toward proinflammatory phenotype. Consistent with the results of TLR4 expression, supplementation with EPs effectively inhibited microglia hyper-activation (Figure 5J-K; Figure S7A-C). Collectively, these results suggest that EPs significantly ameliorated OD-induced hippocampal neuroinflammation in adolescent mice, potentially through inhibition of the LPS-activated TLR4/ NF-κB pathway.

### EPs improved adult neurogenesis deficits induced by OD during adolescence

**Figure 6:**
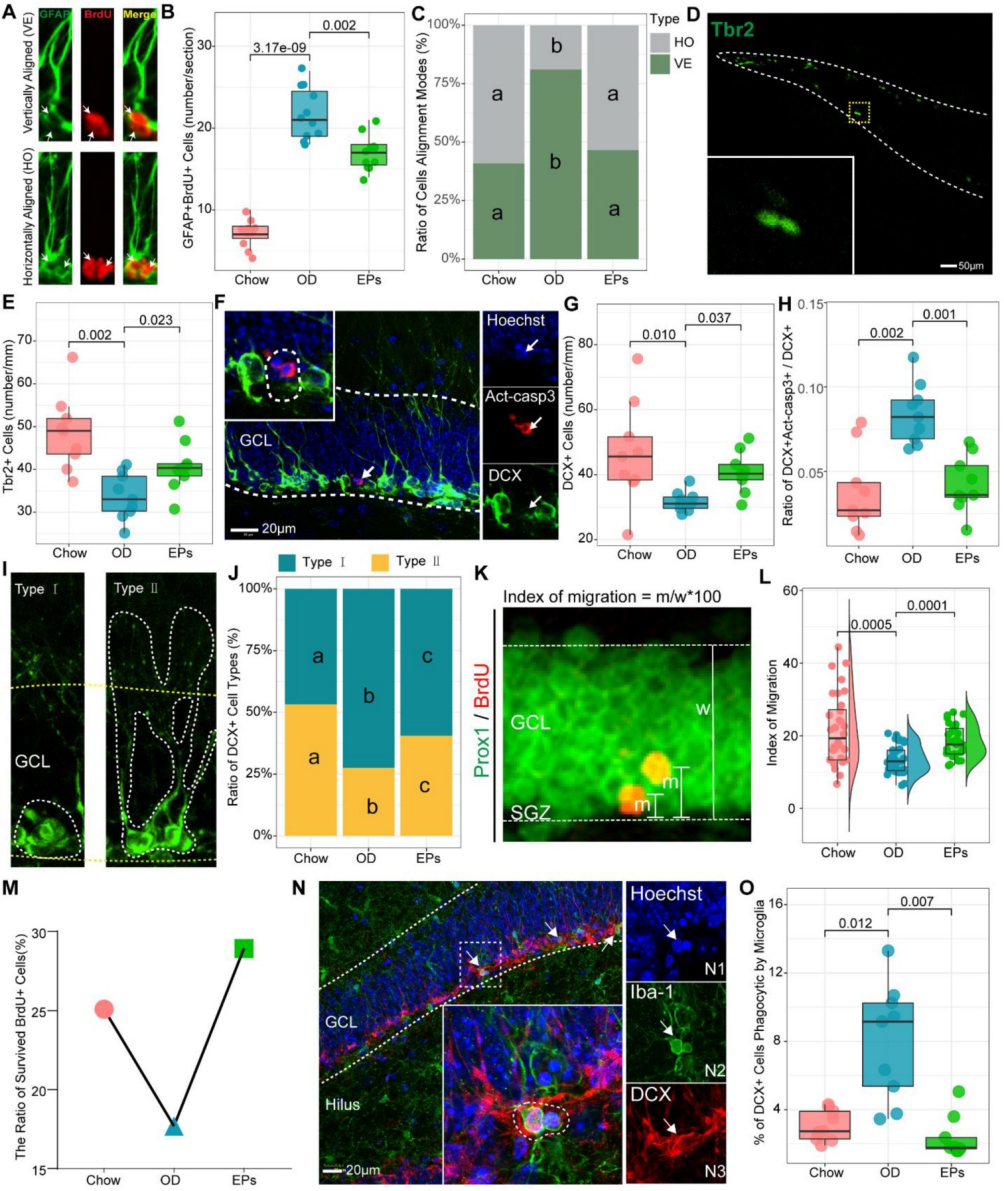
** EPs alleviated the OD-associated deficits of adult neurogenesis in the hippocampus.** (A) Representative pictures of vertically and horizontally aligned BrdU (red) and GFAP (green) double-labeled RGL progenitor cells in SGZ of dentate gyrus. (B) Quantitative analysis of the number of BrdU-labeled RGL progenitor cells in SGZ (*n* = 11 slices from 3 mice). (C) Quantitative analysis of the cell alignment modes in three groups (*n* = 11 slices from 3 mice). (D-E) Confocal photomicrographs and Quantification of Tbr2^+^ progenitor cells in SGZ (*n* = 9 slices from 3 mice). (F) Confocal photomicrographs of double labeling of act-casp3 (red) and DCX (green), a marker for immature neurons. Nuclei were counterstained with Hoechst (blue). (G) Quantification of total DCX^+^ cells (*n* = 9 slices from 3 mice). (H) The percentage of DCX and act-casp3 double-labeled cells in DG significantly increased in OD-mice compared to that in Chow, indicating more newly generated immature neurons went to apoptosis. *n* = 11 slices from 3 mice. (I) Based on morphology, DCX-positive immature neurons can be divided into two subtypes. Type Ⅰ of DCX^+^ cells with processes short than the height of GCL, the younger neurons. Type Ⅱ of DCX^+^ cells with longer processes into the molecular layer, the differentiated neurons. (J) Distribution of two types of DCX^ +^ cells across three groups. The percentage of type I DCX-positive neurons in OD was significantly higher than that in Chow, indicating more newly generated neurons remained in differentiating stage in OD. (K) Images depicting the migration of newly generated neurons double-labeled with BrdU (red) and Prox1 (green), a specific marker for dentate granule cells. The migration index was shown in L.* n* = 33 cells from 3 mice. (M) EPs promoted the ratio of survived BrdU^+^ cells in OD-fed mice. (N) Phagocytosis by microglia (green) involves a ball-and-chain structure formed by microglial terminal branches clearly distinguishable from the microglial cell body (N2). The microglial phagocytic pouch is shown in detail: a pyknotic nucleus (N1) located in the body of DCX^+^ cells (N3) is undergoing phagocytosis. Nuclei were counterstained with Hoechst (blue). (O) The percentage of DCX^+^ cells phagocytic by microglia (*n* = 9 slices from 3 mice). In C and J, the same lower letter indicates no significant difference. Statistical significance compared to OD group by one-way ANOVA, adjusted for multiple comparisons by Dunnett post-hoc test. Lines in boxes represent median, top and bottom of boxes represent first and third quartiles, and whiskers represent 1.5 interquartile range.

Accumulating evidence demonstrates that increased inflammatory responses in the peripheral and CNS negatively affect adult neurogenesis in the hippocampus 46. In addition, research has shown that newly generated neurons are involved in cognitive and social brain functions and that defects in adult neurogenesis result in various brain disorders 47. Thus, we further examined the adult neurogenesis in the hippocampus of mice with or without OD. We found that OD induced a marked increase in type-1 cells, double-labeled with GFAP and 5-Bromodeoxyuridinc (BrdU) and with radial glialike morphology in the dentate gyrus (DG) (Figure 6A-B), suggesting that OD disturbed the quiescence maintenance of radial neural stem cells (rNSCs). Quiescence is a universal protective mechanism that counteracts the depletion of stem cells in basal conditions, preserving the ability to meet local physiological and pathological tissue needs throughout life 48. EPs supplementation inhibited the over-activation of the rNSCs in OD-fed mice (Figure 6B). Neurogenesis is explained by multiple modes of cell division. Asymmetric cell division results in one cell differentiating into neurons while the other retains rNSC properties. Symmetric cell division generates two cells both maintaining stemness, which extends the progenitor cell pool 49. To investigate the effect of OD on the cell alignment modes, we focused on the two-nucleus cells with BrdU^+^/GFAP^+^ (Figure 6A). Our results showed that OD predominantly stimulates the asymmetric division of rNSCs in the subgranular zone (SGZ), indicating that OD during adolescence may induce the depletion of stem cells pool in SGZ, which was mitigated by EPs supplementation (Figure 6B-C).

**Figure 7:**
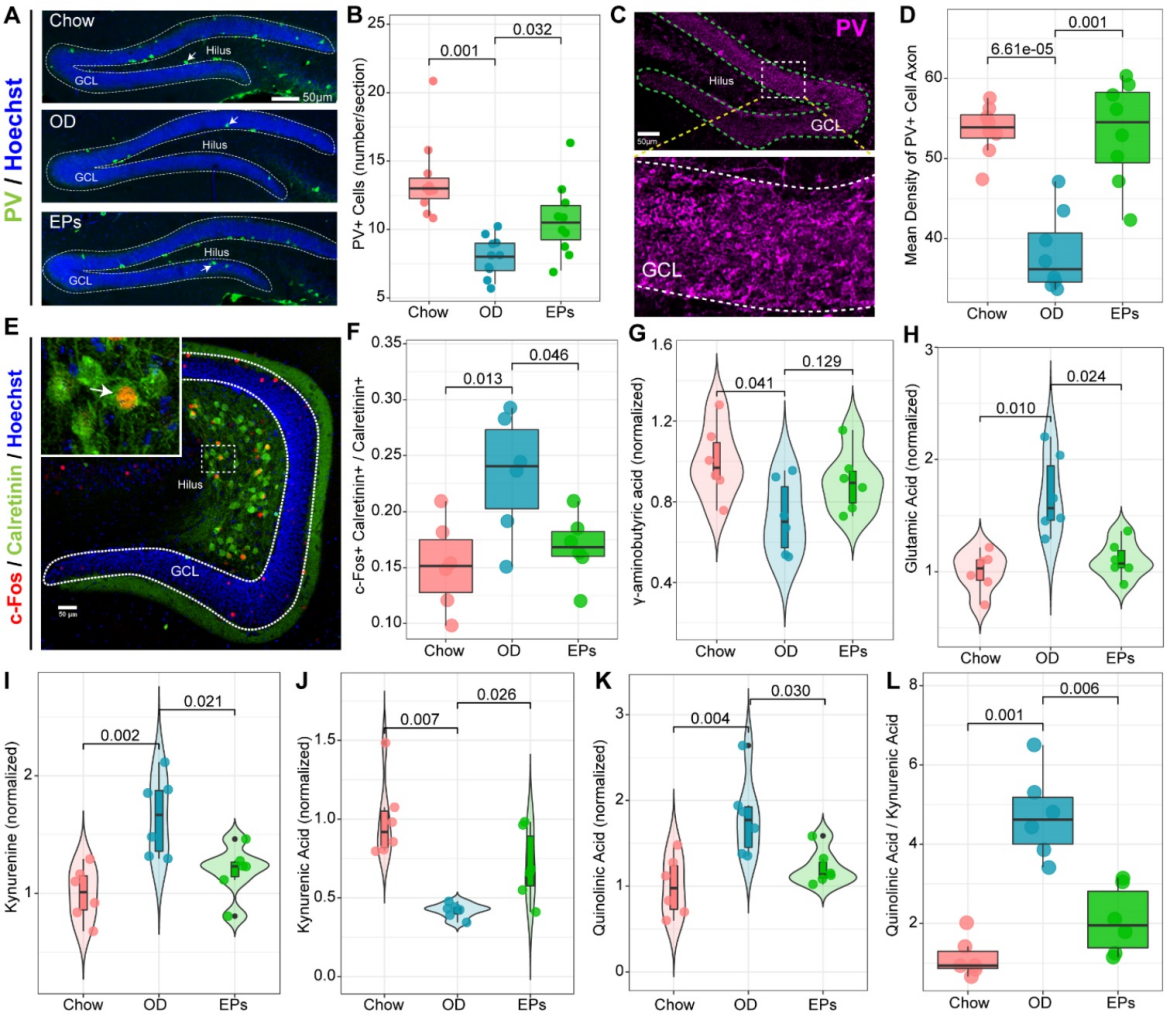
** EPs inhibited the Kyn pathway and rebalanced GABA and glutamate acid in the hippocampus of OD-fed mice.** (A) Confocal images of dentate gyrus with immunohistochemistry for Parvalbumin (PV), a specific marker for one subset of GABAergic interneurons. Nuclei were counterstained with Hoechst (blue). The somata of a majority of PV-positive interneurons (green) were located in the SGZ of DG. (B) Quantification showed that compared to that in DG of Chow, the number of PV-positive interneurons in DG of OD significantly decreased, which was prevented by supplementation of EPs. *n* = 9 slices from 3 mice. (C) Representative high magnification image of PV-positive axonal projection in granule cell layer of DG. (D) Quantitative analysis showed that EPs prevented the DG from reducing PV-positive axonal projections in granule cell layer induced by OD. *n* = 9 slices from 3 mice. (E) Representative confocal image of double immunohistochemistry with antibodies against c-Fos (red) and Calretinin (green), a marker of mossy cells in DG. The nuclei (blue) of many Calretinin-positive mossy cells were co-labeled with c-Fos, indicating the activation of mossy cells. (F) Quantification of mossy cells double-labeled with c-Fos and Calretinin in the DG showed that OD induced the overactivation of mossy cells compared to Chow, which was rescued by supplementation of EPs (*n* = 6 slices from 3 mice). Gas chromatography-mass spectrometry was used to assess the concentration of GABA (G), glutamic acid (H), kynurenine (I), kynurenic acid (J), quinolinic acid (K), and the ratio of quinolinic acid/kynurenic acid (L) in the hippocampus (*n* = 6 individuals/group). Compared to Chow, OD significantly reduced the inhibitory neurotransmitter GABA, whereas the excitatory neurotransmitter glutamate increased, resulting in an imbalance of inhibition and excitation of hippocampal circuits. Supplementation of EPs mitigated this imbalance. Statistical significance compared to OD group by one-way ANOVA, adjusted for multiple comparisons by Dunnett post-hoc test. Lines in boxes represent median, top and bottom of boxes represent first and third quartiles, and whiskers represent 1.5 interquartile range.

Recent studies demonstrate that mossy cells (MCs) and interneurons in DG act as key niche components to control the activation versus quiescence of the rNSCs mediated by the γ-aminobutyric acid (GABA) and glutamate receptors expressed in rNSCs, respectively 50. To determine the activation state of MCs in the hippocampus, we performed double-immunostaining with antibodies against c-Fos and Calretinin, a marker for MCs. We observed that OD significantly increased the density of c-Fos+ MCs in the hippocampus (Figure 7E-F). Meanwhile, targeted metabolomics analysis showed that the level of glutamic acid was significantly increased in the hippocampus of OD-fed mice (Figure 7H), suggesting that OD significantly enhanced the activation of MCs, breaking the balance of rNSCs maintenance and neurogenesis which is essential to ensure continuous neuronal generation in the hippocampus throughout life without depleting the rNSCs pool. Moreover, the density of PV-positive interneurons and axons also decreased in OD-fed mice (Figure 7A-D). These results were consistent with GABA reduction in hippocampal tissues (Figure 7G). Song et al. revealed that rNSCs respond to GABA, and only the PV-positive GABAergic interneurons exerted an effect on rNSCs quiescence 51. These results indicated that OD impaired PV^+^ interneurons-related loops, which exacerbated hyperactivation of rNSCs.

The gut microorganisms affect the structure and function of brain and are engaged in neuropsychiatric disorders, in part by regulating Trp metabolism 41. Kynurenic acid (Kna) and QA are generated by astrocytes and microglia in the brain, respectively, and exert different effects on neurons by decreasing or increasing extracellular levels of glutamate, a major excitatory neurotransmitter in CNS 52. As a result of the enhanced Kyn pathway in serum of OD-fed mice (Figure 4D), we further examined the Kyn metabolic pathway in CNS. Consistent with the hyperactivation of hippocampal microglia, OD significantly enhanced the concentration of Kyn, QA, and the QA/Kna ratio in the hippocampus (Figure 7I, K-L). Supplementation with EPs notably inhibited Kyn pathway in the hippocampus of OD-fed mice, including reduced Kyn, QA, and QA/Kyn ratio and increased Kna (Figure 7I-L). These results well explain the increased glutamic acid level in the hippocampus (Figure 7H) and the excessive activation of MCs (Figure 7E-F) in OD-fed mice, suggesting that EPs supplementation effectively mitigated the hyperactivation of rNSCs in OD-fed mice, possibly due to the inhibition of QA-related increases in extracellular glutamate.

One of the important issues in adult hippocampal neurogenesis is the survival of newly generated neurons 53. Inconsistent with the hyperactivation of rNSCs, the number of activated intermediate progenitor cell type (Type 2a/2b), expressing Tbr2, and neuroblast (Type 3), expressing DCX, was decreased in OD-fed mice (Figure 6D-G). Fate choice and survival of newborn neurons are significantly regulated by multiple modulatory neurotransmitters. The effect of EPs in preventing the decline of intermediate progenitor cells and neuroblasts in SGZ (Figure 6D-G) may result from inhibiting the over-activation of mossy cells (Figure 7E-F) and promoting the functional integrity of PV^+^ cells (Figure 7A-D), thereby rebalancing the GABAergic and glutamatergic transmission in hippocampus. Consistent with the above results, EPs remarkably reduced the apoptotic DCX^+^ immature neurons (Figure 6F, H) and promoted the mature of neuroblast (Figure 6I-J) in DG of OD-fed mice. These apoptotic immature neurons are mainly phagocytosed by microglia, which form a phagocytic pouch that engulfs the apoptotic cell (Figure 6 N2). Unsurprisingly, OD significantly increased the ratio of phagocytosed DCX^+^ immature neurons by microglia, which was inhibited by EPs supplementation (Figure 6N-O).

Newborn neurons in the hippocampus are required to migrate and functionally integrate into the granule cell layer (GCL) of the DG to perform physiological functions, and this process lasts for at least four weeks 54. To assess the impact of OD on the migration of newborn neurons in the DG of adolescent mice, BrdU was administered to the mice for three consecutive days before the start of the experiment. The advantage of this experimental strategy is that we were able to assess the survival and migration of newly generated neurons in the DG during the entire experiment. We observed that EPs enhanced the ratio of survived newborn cells in OD-fed mice (Figure 6M). Adult newborn neurons in SGZ migrate only a short distance into the DG 54, and neurotransmitters dysfunction 55 and neuroinflammation 56 significantly disturb this migration pattern. In line with the above results, OD during adolescence significantly disturbed the migration of newborn neurons in the DG, which was rescued by supplementation with EPs (Figure 6K-L).

## Discussion

**Figure 8:**
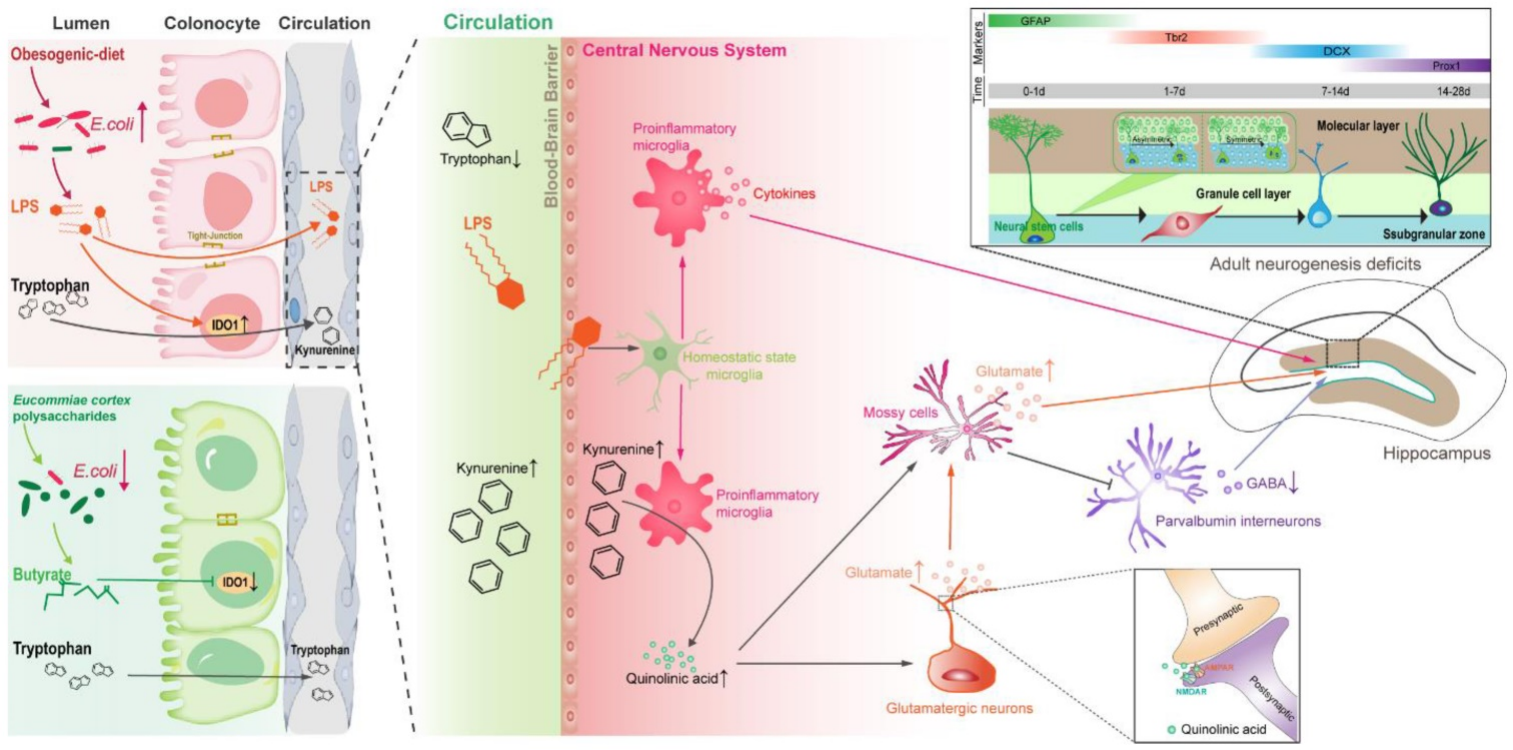
** Proposed mechanism for EPs mitigated OD-induced behavioral dysfunction.** Persistent OD during adolescence reduced the abundance of SCFAs-producing bacteria but thrived facultative anaerobic bacteria, thereby increasing the endotoxin concentrations in colon lumen and circulation. In OD-fed mice, the IDO1 enzyme was activated in colonic cells, upregulating the peripheral Kyn pathway which promoted the production of Kyn and its downstream metabolites, including QA and Kna in the blood and brain parenchyma. Furthermore, infiltrating endotoxin stimulated the microglia toward proinflammatory phenotype which directs the metabolism of Kyn toward QA and increased extracellular glutamate levels in the hippocampus. QA-induced imbalance of GABAergic and glutamatergic transmission and microbiota-derived LPS induced neuroinflammation led to adult neurogenesis defects in the hippocampus and impaired hippocampus-dependent social and cognitive function in adolescent mice. EPs supplementation promoted the growth of butyrate-producing microbes and the production of butyrate and inhibited the abundance of *E.coli*, thereby alleviating OD-induced gut dysbiosis and peripheral Kyn pathway. This contributed to mitigating the QA-related neurotransmitter dysfunction and endotoxin-triggered neuroinflammation, thereby remodeling the rhythm of hippocampal neurogenesis and improving behavioral dysfunction in OD-fed mice.

Adolescence is a critical developmental period and vulnerability to the onset of psychiatric diseases 57. Using a mouse model, we demonstrated that OD during adolescence is a potential risk factor for the development of behavioral phenotypes associated with cognitive and social dysfunction. Although previous pharmacological experiments have shown that EPs possess neuroprotective and anti-inflammatory activities, the effects of polysaccharides on OD-induced gut dysbiosis and the following inflammation had not been investigated. We observed that EPs supplementation improved the obesity-related symptoms and protected the OD-fed mice against behavioral dysfunctions, possibly by remodeling the gut microbiome and attenuating OD-induced disorders of Trp metabolism (Figure 8).

A causality between OD and gut dysbiosis has been well established. The specialized anaerobic microorganisms that reside in our colon are able to translate complex dietary fiber into SCFAs that support host nutrition, immune development, and pathogen restraint 37. In comparison, facultative anaerobic bacteria, such as *Enterobacteriaceae*, do not confer such benefits, but endotoxin 58, 59. A recent human cohort study revealed that the human gut microbiota within 24 hr after reducing fiber intake to 30 g/day was dramatic and rapid rearrangement, accompanied by a reduction in the production of SCFAs in the colon 60. In the present study, four weeks of OD significantly altered the gut bacterial community, especially reducing the abundance of butyrate-producing bacteria. Butyrate directs the metabolism of surface colonocytes toward mitochondrial β-oxidation of SCFAs, which helps to maintain an anaerobic environment in the lumen of colon, thus inhibiting the growth of facultative anaerobic bacteria and maintaining intestinal homeostasis 37. In this study, we observed that OD remarkably reduced the butyrate level in the colon and increased the abundance of *E.coli*, a microbial signature of intestinal dysbiosis. Meanwhile, SCFAs also contribute to the suppression of intestinal inflammation. For example, butyrate can inhibit the transcription of pro-inflammatory genes, including TNF-α and IL-1β 9. Therefore, the beneficial effects of EPs supplementation on gut dysbiosis may be attributed to the promotion of butyrate-producing bacteria and the restoration of intestinal immune homeostasis. Notably, elucidating the response mechanisms of the gut microbiota is challenging due to the complexity and high variability of the gut microbial structure. Although we observed the beneficial effects of EPs on gut microbes, more evidence is needed to elucidate the underlying mechanisms. Previous studies have highlighted the initiating role of gut microbiota in OD-induced obesity-related symptoms, including weight gain and systemic inflammation. Therefore, prebiotic-based gut bacteria interventions are considered to possess great potential for the prevention and therapy of diet-related impairments. Consistent with the remodeling effects on gut microbiota, EPs coadministration effectively mitigated OD-induced obesity-related symptoms, including weight gain, elevated serum cholesterol, and epididymal fat accumulation.

Extensive evidence from rodent and human studies suggests that OD consumption is associated with impaired hippocampus-dependent learning and memory function 61. However, the effects of OD on behavioral phenotypes in adolescence had rarely been reported, especially the underlying mechanisms. Growing evidence demonstrates that OD affects brain function through the gut-brain axis 62. Bruce-Keller et al. showed that transplanting feces from OD-fed adult mice to mice pretreated with antibiotics fed a controlled diet increased anxiety and decreased memory 10, suggesting that gut microbiota is essential in mediating OD-related cognitive and social dysfunction. The DG in the hippocampus is one of two regions in which adult neurogenesis 63. Adult neurogenesis is a developmental process that generates functionally integrated neurons from rNSCs and occurs throughout life in the hippocampus of the mammalian brain 63. This process relies on a range of environmental and cellular factors and is also governed by the activity of local neural loops 46 and is recognized to dramatically shape social and cognitive function 54. In the present study, we found that OD during the adolescent period induced the hyperactivation of rNSCs and led to a strong tendency for asymmetric division of rNSCs. Moreover, OD significantly impaired the survival and migration of newborn neurons. Disentangling adaptive and maladaptive responses will contribute to the understanding of disease development. However, the results of this study do not support the well understanding of these changes. Glutamatergic and GABAergic signaling has been demonstrated to be essential for the activated rNSCs to select an appropriate fate during the first week and for newborn neurons to compete for synaptic integration and promote survival 64, 65. Mossy cells, one of the major glutamatergic neurons in DG, provide the first glutamatergic input to newly generated neurons starting from 5 to 10 days after birth 66. EPs supplementation ameliorated OD-induced adult neurogenesis deficits may attribute to rebalancing the GABAergic and glutamatergic transmission in hippocampus.

Investigations of the gut-brain axis have shown that the gut microbiota plays a crucial role in coordinating brain development and behavior and that microbiota-mediated metabolites serve as a crucial regulator of these interactions. Metabolomic analysis revealed that intestinal microbes affect host metabolism and immunity through a variety of chemically distinct metabolites, including amino acid metabolites 67. Any disruption in the host-microbiota cascade may be a factor in the initiation or intensification of disease pathogenesis. Disruption of microbiota composition and function caused by lifestyle preferences has been implicated as a crucial contributor to metabolic diseases 2, 41. In the present study, our results revealed that OD disrupted Trp metabolism in adolescent mice. Trp, an essential aromatic amino acid, has gained increasing attention in a range of metabolites at the interface between the gut microbiota and the host 41. As Trp is not generated from animal cells, humans are dependent on exogenous, mainly dietary, intake 41. Three major Trp metabolic pathways resulting in 5-hydroxytryptamine, Kyn, and indole derivatives are regulated by the gut microbiota 41. A tiny fraction of ingested Trp is consumed for anabolic processes, while most of it is metabolized through the Kyn pathway of Trp degradation. Metabolites in the Kyn pathway are thought to play an important role in neurodegenerative disorders 52. The tissue-specific expression of enzymes in the Kyn pathway is best studied for IDO1 68. The IDO1 enzyme is highly expressed in inflamed tissues, with its expression induced by the proinflammatory cytokine IL-6 and TLR ligands 68. OD-induced dysbiosis led to immune dysfunction in the colonic tissues and increased the concentration of LPS in the colon lumen, resulting in enhanced expression and activity of IDO1 in colonocytes. This process contributed to the upregulation of the peripheric Kyn pathway in OD-fed mice. The CNS receives approximately 60% of Kyn from the periphery, and thus Kyn concentrations in the CNS are significantly influenced by peripheral factors 69. Kyn and its metabolites, QA and kynurenic acid (Kna), are well known due to their impact on the CNS and are associated with several psychiatric and psychological disorders 70. As Trp and Kyn easily cross the blood-brain barrier, it is clear that fluctuations in the blood levels of these metabolites directly affect metabolism in the Kyn pathway - including the synthesis of Kna and QA - in the brain 40. Kna and QA exert different effects on neurons by decreasing or increasing extracellular levels of glutamate. The commissural fibers derived from MCs provide direct glutamatergic and indirect GABAergic inputs to rNSCs. The indirect GABA pathway dominates and promotes rNSC quiescence when MCs are activated at moderate level, whereas the direct glutamate pathway dominates and promotes rNSC activation when MCs are activated at high level 50. We observed significantly enhanced concentration of Kyn and QA in the CNS of OD-fed mice, which increased the level of glutamic acid in the hippocampus and overactivated MCs, resulting in adult neurogenesis defects. Due to the intricacy of the adult neurogenesis, the transmitter and loop pathways may not fully reveal the mechanisms driving the impairment of adult neurogenesis in OD mice. Notably, EPs supplementation attenuated dysbiosis-related metabolic syndrome and restored the rhythm of adult neurogenesis in adolescent mice fed by OD, alleviating OD-induced social and cognitive behavior abnormalities.

Rodent studies consistently indicate that long-term consumption of OD raises levels of neuroinflammatory markers 61. The current model of OD-induced chronic inflammation is mainly explained by gut dysbiosis and increased concentration of serum LPS, a condition called metabolic endotoxemia 2. Low concentrations of LPS in the blood may cause systemic and targeted inflammation, such as neuroinflammation, in OD-fed mice and obese humans through activation of TLR4 signaling in various cells 38. Thus, we believe that remodeling gut dysbiosis is crucial to alleviating OD-related systematic inflammation. In the present study, we observed that OD during adolescence was sufficient to result in hippocampal microglial M1 polarization, a sign of neuroinflammation. Recent studies suggest that early-life inflammation causes hyperactivation of microglia, promoting the development of depression-like symptoms during adolescence 71. Moreover, clinical reports implicate a positive association between circulating inflammatory factors and cognitive decline in humans 72. Consistent with the remodeling effect of EPs on gut microbiota, EPs supplementation improves gut barrier integrity, reduces serum level of endotoxemia, and suppresses the subsequent hippocampal neuroinflammation in OD-fed mice. In addition, microglia also plays a crucial role in regulating Trp metabolism in the CNS. The reason is that neuroexcitatory QA is produced by microglia, and the activated microglia in the inflammatory disease will intensify QA production and induce neuroexcitotoxicity 40. Our results indicate that EPs mitigated cognitive and social dysfunction by partly inhibiting gut dysbiosis-mediated neuroinflammation. While we found that EPs significantly inhibited the expansion of *E. coli* in the intestinal lumen, the regulatory mechanism still needs to be further explored.

Taken together, our results revealed that OD during adolescence resulted in individual cognitive and social dysfunctions by inducing gut dysbiosis and upregulating the Kyn pathway in the periphery and CNS. These results offer a more in-depth understanding of the negative effect of OD, especially on adolescence. Notably, oral EPs reshaped the gut microbial composition, inhibiting colitis and alleviating the following metabolic syndrome. Meanwhile, EPs effectively suppressed the OD-induced neuroinflammation and the deficits of adult neurogenesis, thereby restoring abnormal neurocognitive and social behaviors in OD-fed adolescent mice. Collectively, these findings open new research avenues into preemptive therapies for neurodevelopmental disorders that target the OD-induced gut dysbiosis and indicate that EPs may be used as prebiotics to restore microbial community and subsequently associated deficiencies.

## Supplementary Material

Supplementary figures and table.

## Abbreviations

EPs: *Eucommiae cortex* polysaccharides
OD: obesogenic diet
CNS: central nervous system
SCFAs: short-chain fatty acids
RF: Random forest
PCoA: performing principal coordinates analysis
LPS: lipopolysaccharide
TNF-α: tumor necrosis factor α
IL-1β: interleukin-1β
MCP-1: monocyte chemoattractant protein-1
WGCNA: weighted gene co-expression network analysis
Trp: tryptophan
Kyn: kynurenine
Kna: kynurenic acid
IAA: indole acetic acid
IPA: indole-3-propionic acid
IDO1: indoleamine 2, 3 dioxygenase1
QA: quinolinic acid
iNOS: inducible nitric oxide synthase
IL-6: interleukin- 6
rNSCs: radial neural stem cells
GFAP: glial fibrillary acidic protein
BrdU: 5-Bromodeoxyuridinc
MCs: mossy cells
GABA: γ-aminobutyric acid
Tbr2: T-brain gene-2
DCX: doublecortin
GCL: granule cell layer
DG: dentate gyrus
SGZ: subgranular zone
TLR4: toll-like receptor 4
*E.coli*: *Escherichia coli*
ASVs: amplicon sequence variants
Iba1: ionized calcium binding adapter molecule 1
PV: parvalbumin
