# Supplementary material for: Eucommiae cortex polysaccharides mitigate obesogenic diet-induced cognitive and social dysfunction via modulation of gut microbiota and tryptophan metabolism
Source: Theranostics. 2022 May 1;12(8):3637–55. doi: 10.7150/thno.72756 (PMC9131264; doi:10.7150/thno.72756)
Supplement: Supplementary file 1 — Supplementary figures and table. [file thnov12p3637s1.pdf]

Supplementary Information

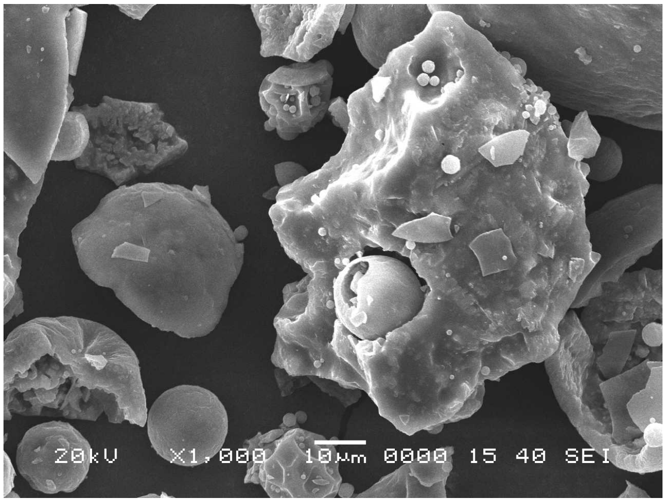

Figure S1. Scanning electron microscope image for EPs.

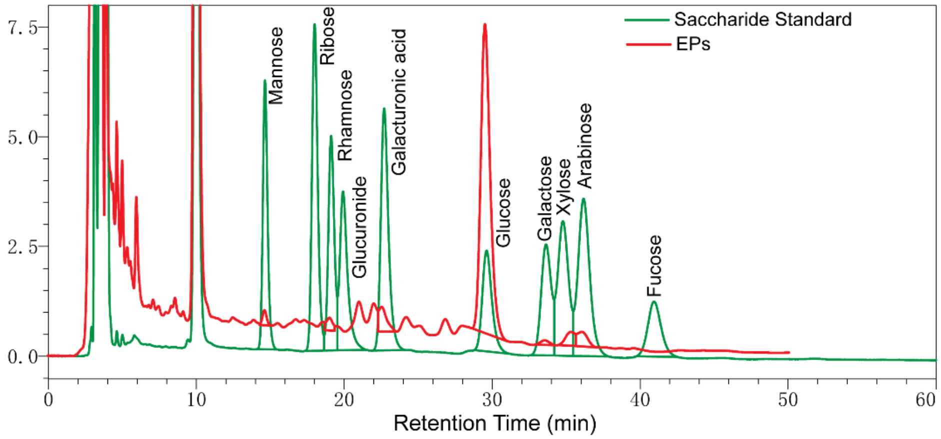

Figure S2. The elution profile of the saccharide standard and EPs by high-performance liquid chromatography.

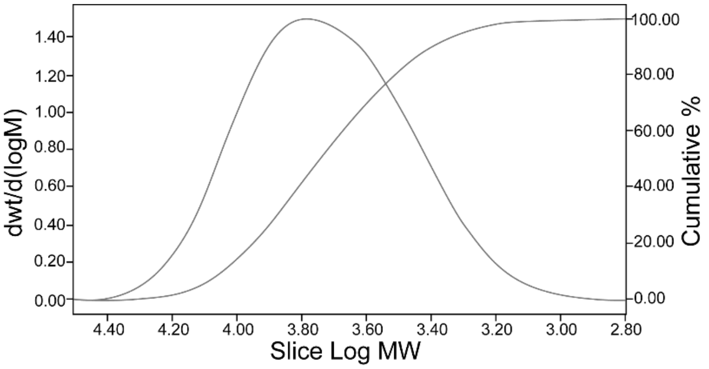

Figure S3. The molecular weight distribution of EPs.

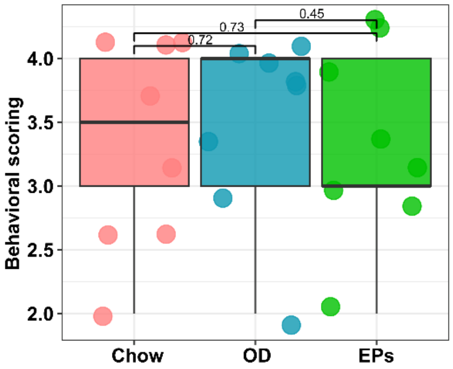

Figure S4. Behavioral scoring of the rotarod test (n = 8 individuals/group). Statistical significance compared to each group by one-way ANOVA, adjusted for multiple comparisons by Dunnett post-hoc test.

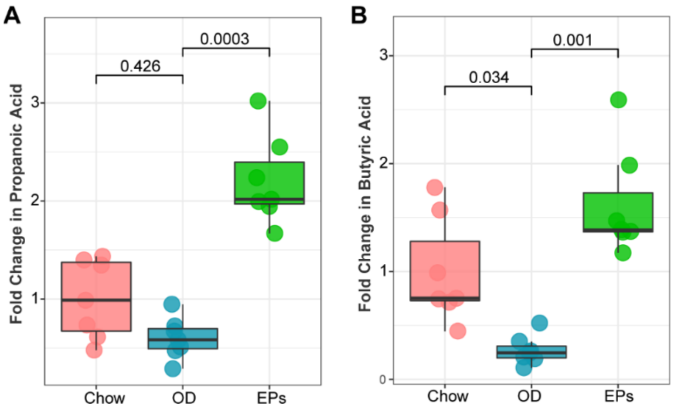

Figure S5. Quantitative analysis of SCFAs concentration, including propanoic acid (A) and butyric acid (B), in colon contents from three groups (n = 7 individuals/group). Statistical significance compared to OD group by one-way ANOVA, adjusted for multiple comparisons by Dunnett post-hoc test.

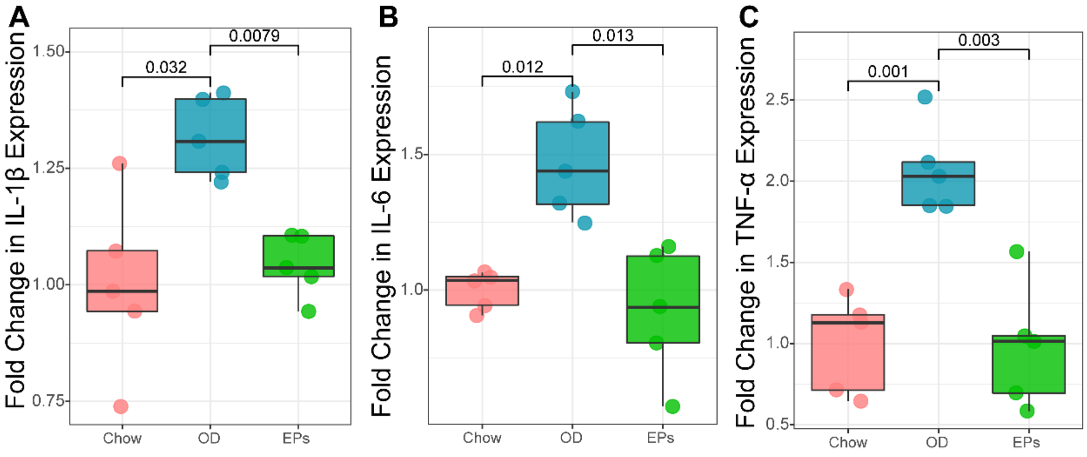

Figure S6. Concentrations of three representative proinflammatory cytokines, IL-1β (A), IL-6 (B), and TNF-α (C) in the hippocampus (n = 5 individuals/group). Statistical significance compared to OD group by one-way ANOVA, adjusted for multiple comparisons by Dunnett post-hoc test.

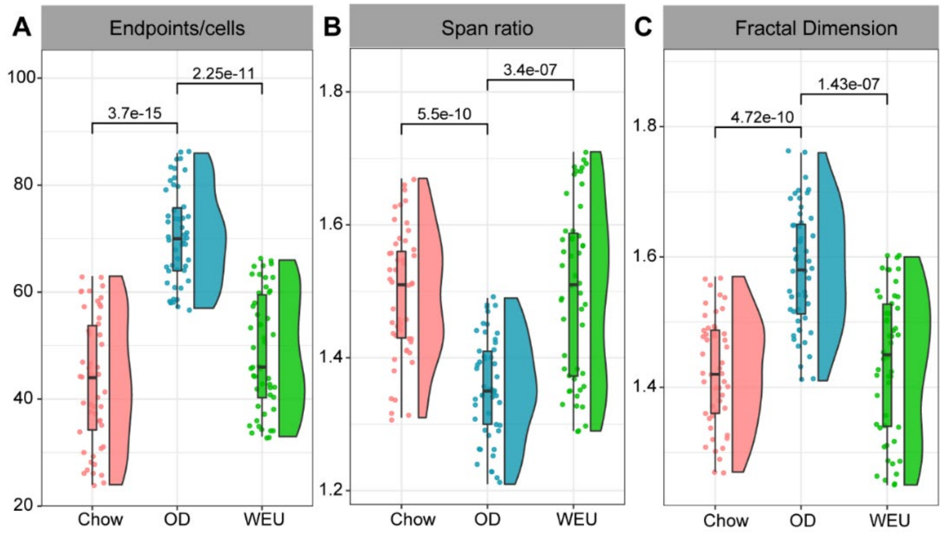

Figure S7. Morphological analysis of microglia.

Summary data and statistical analysis of endpoints (A), span ratio (B), and fractal dimension (C) between three groups. *n* = 50 cells from 3 mice. Statistical significance compared to OD group by one-way ANOVA, adjusted for multiple comparisons by Dunnett post-hoc test.

Table S1. The monosaccharide of EPs.

Monosaccharide (% w/w)	
Mannose	1.12872
Rhamnose	2.18416
Galacturonic acid	3.15037
Glucose	82.70771
Galactose	0.79398
Xylose	2.01605
Arabinose	1.99197
